# Treatment of *de novo* femoro-popliteal lesions with a new Drug Coated Balloon: early experience of a single Center in the first 50 patients

**Published:** 2019-01-20

**Authors:** UM Bracale, M Di Filippo, A De Capua, L Vanni, D Narese, F Pecoraro, AM Giribono, R Bracale

**Affiliations:** 1 Vascular Surgery Unit, Department of Public Health, University Federico II of Naples, Naples, Italy; 2 Department of Radiology, University of Campania ‘Luigi Vanvitelli’, Naples, Italy; 3 Vascular Surgery Unit, University of Palermo, Palermo, Italy; 4 Department of Medicine and Health Science, University of Molise, Campobasso, Italy

**Keywords:** drug-coated balloon, peripheral arterial disease, superficial femoral artery, endovascular treatment

## Abstract

Angioplasty with drug-coated balloon (DCB) is an emerging and reliable method for the treatment of femoro-popliteal lesions. We report our experience with the Stellarex™ DCB in the first 50 patients.

**Methods:**

From July 2015 to November 2017, 50 patients (41 M, 9F), medium age (64 ± 7.4 year) were subject to 33 angioplasties (PTAs) for femoro-popliteal lesions with a paclitaxel-coated balloon (Stellarex™). Based upon clinical data sixteen patients had severe claudication (56% - Rutherford class 3); ten patients suffered from ischemic rest pain (34% - Rutherford class 4); and five presented minor tissue loss (10% - Rutherford class 5). 42% of patients showed femoro-popliteal lesion TASC-II B, and 58% presented lesions pertaining to TASC-II C.

**Results:**

Immediate technical success was 100% without perioperative complications. Primary patency rate was 94% at twelve months. In three cases restenosis (6%) was detected within a year from procedure, and a further PTA DCB was performed with primary assisted patency rates of 100% at twelve months. Two patients underwent major lower limb amputation. Three patients died during follow-up and one patient was lost at follow-up.

**Conclusion:**

DCB angioplasty with Stellarex™ is a viable alternative to traditional endovascular procedures proving satisfactory primary patency rates at twelve months. Based on our experience, treatment with DCB is a first choice technique for non-complex *de novo* lesions of the femoro – popliteal tract.

## I. INTRODUCTION

In peripheral artery disease, endovascular treatment with percutaneous transluminal angioplasty is considered a primary option however probability of restenosis within the first 6 – 12 months after treatment (1,2) is higher than with stenting or drug coated-balloons as demonstrated in a number of previous trials (3–6). Balloon inflation can cause an injury in the vascular wall, which may subsequently trigger immediate elastic recoil, intimal dissection and negative vascular remodeling by neointimal hyperplasia. Nitinol stenting can prevent elastic recoil and dissection and risk of early occlusion thereafter, however it cannot inhibit neo-intimal hyperplasia. Treatment with drug-eluting stents is surely a promising improvement yet presents intrinsic limits on stent implantation such as the presence of a foreign body, risk of fracture and difficult treatment in cases of in-stent restenosis. Drug Coated Balloon (DCB) provides the possibility to treat target lesions by way of local drug release with no foreign bodies left behind. Given these advantages, the purpose of our study was to assess short and medium term effectiveness as well as safety of DCB treatment.

## II. METHODS

Our study was a retrospective, single – arm and single center study in which patients underwent femoro-popliteal angioplasties with the Stellarex™ coated balloon (Spectranetics Corp., Colorado Springs, CO); a 0.035″ compatible over-the-wire device coated with paclitaxel (2 μg/mm^2^ balloon surface) and polyethylene glycol, an excipient that facilitates drug transfer into the vessel wall. From July 2015 to November 2017, 50 patients (41 M, 9F) were subject to 53 PTA DCBs for femoro-popliteal lesions. All patients had the age >18 years, life expectancy>1 year and were affected by lower limb ischemia classified as Rutherford class 3, 4 and 5. The angiographic criteria included *de novo* lesion >70% stenosis within the superficial femoral artery and/or popliteal artery down to the trifurcation, target reference vessel diameter 4 to 6 mm, patent inflow artery and at least one patent tibioperoneal run-off vessel (7). According to the American Heart Association practice guidelines, the restenosis was defined as the focal systolic peak velocities (PSV)>300 cm/s, velocity ratio (Vr)>3.0, and uniform PSVs <50 cm/s (8). In forty-eight cases (85.7%) only an angioplasty with DCB was performed. Eight cases required additional procedures: four stents were deployed in the superficial femoral artery following DCB angioplasty PTA; two iliac stenting and two femoral endarterectomies were also necessary. After treatment, all patients underwent dual antiplatelet therapy (aspirin + clopidogrel) for six months. Follow-up included: clinic exam, Ankle-Brachial Index (ABI) and duplex scan at 1, 3, 6, 12 months and annually thereafter.

## III. PROCEDURE

Three days prior to procedure patients received dual antiplatelet therapy, while a single dose of 3500 U.I. of sodium heparin was administered intravenously upon placement of the long sheath during procedure. In forty-eight cases the procedures were performed under local anesthesia; in two cases, spinal anesthesia was given in order to perform a femoral endarterectomy. A retrograde contralateral femoral approach with a long reinforced sheath (6 Fr 45 cm, Destination - Terumo, Tokyo, Japan) was chosen in order to give more support and to perform selective angiography. Target vessel recanalization was carried out with a Berenstein or a Vertebral 4 Fr catheter and a standard soft 0.035″ guidewire or a 0.018″ guidewire trying to remain within the true lumen whenever possible. A predilation of the target lesion was performed in all patients with an uncoated balloon 1 mm less than the reference vessel diameter to avoid drug dispersion during the lesion crossing. Size of the DCB was determined according to the diameter of the vessel based on preoperative ultrasound images in a one to one ratio. Inflation time of the DCB was at least 3 minutes.

## IV. FOLLOW-UP AND ENDOPOINTS

Clinical evaluations were made at 1, 3, 6 and 12 months post-procedure with a Duplex ultrasound of the target lesions, ABI, and evaluation if any adverse events were present. Endpoints were primary patency and freedom from major adverse events (MAE) defined as limb amputation, death, cardiovascular accident and restenosis at 12 months.

## V. STATISTICAL ANALYSIS

Statistical analysis was performed using SPSS 20.0 for Windows (IBM Corp, Armonk, NY). Continuous data are expressed as mean ± standard deviation, categorical variables are expressed as counts and percentage while Kaplan – Meier estimates are presented for patency.

## VI. RESULTS

**Fig. 1:**
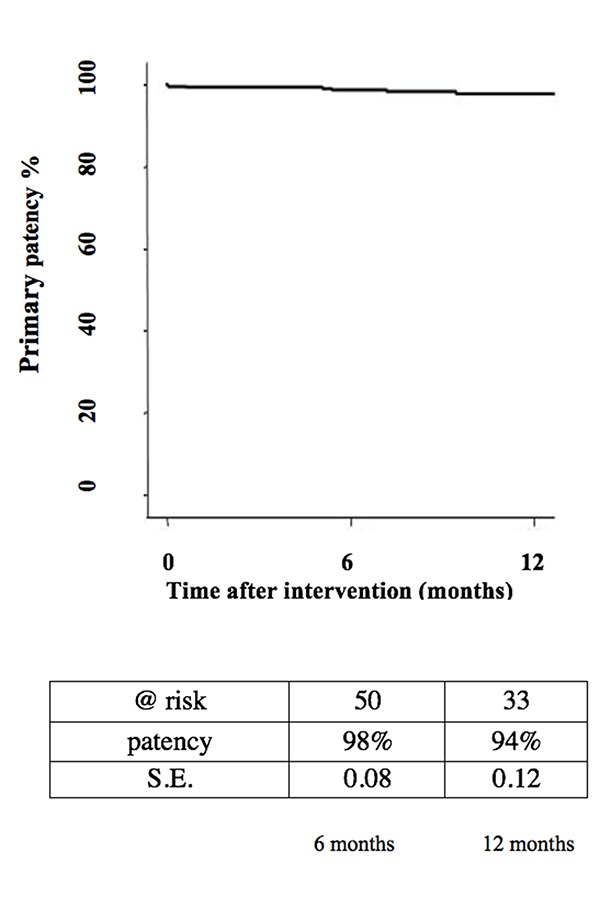
94% primary patency rate at 12 months

Mean age was 64 ± 7.4 years. Twenty-nine (58%) of patients were currently smokers, 66% had hyperlipidemia, 68% had diabetes, 64% were affected with hypertension, 14% had chronic renal failure, 76% had COPD and 42% CAD. Based on clinical data: twenty-eight patients had severe claudication (56% - Rutherford class 3); seventeen patients suffered from ischemic rest pain (34% - Rutherford class 4); and five presented minor tissue loss (10% - Rutherford class 5). Forty-two percent (42%) of patients showed femoro-popliteal lesion TASC B, 58% presented lesions belonging to TASC C. Lesions were located 1 cm distal to the femoral bifurcation and at least in 1 segment of the popliteal artery (mean length of lesions 89 ± 3.2mm). Angiography showed evidence of from 70 to 90% stenosis or total occlusions (18%). Superficial femoral artery (SFA) was involved in 82% of cases. Fifty-three DCBs were employed in 50 patients (mean length balloons 91.1±33.8 mm; mean balloon diameter 4.9±0.7mm). In two cases two DCBs were used. Primary patency rate at 12 months as shown by Kaplan – Meyer survival estimates was 94% (Fig. 1).

**Fig. 2:**
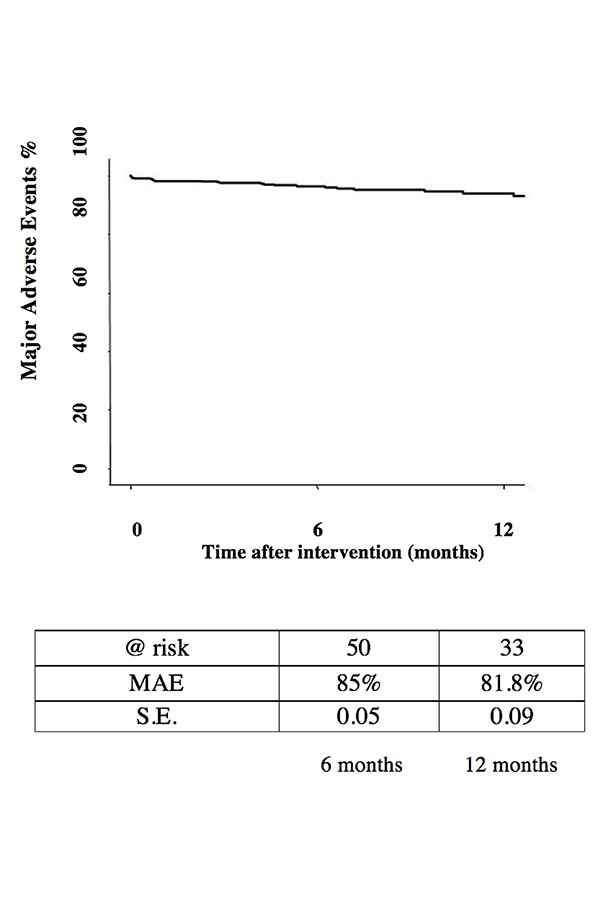
81.8% Freedom from MAE at 12 months

Freedom from major adverse events was 81.8% at 12 months, as shown by Kaplan – Meier (Fig. 2).

The mean ABI increased by 0.35 from preoperative value of 0.59 ± 0.1 to value postoperative 0.94 ± 0.2 at 1 month and remained elevated at 0.94 ± 0.2 at 1 year (p < 0.001). All occlusions were patent at 12 months. Additional procedures included: four lesions requiring a self-expandable stent following DCB due to residual stenosis >50% (ZilverFlex™ - Cook Medical, Bloomington, Indiana, U.S.A.) mean diameter 6±1 mm; middle length 103.3±40.4 mm); two lesions requiring iliac stenting [Dynamic (BIOTRONIK AG · Switzerland) 9×38 mm and ZilverFlex 8×80 mm in common iliac artery and Everflex (Medtronic/Covidien, Mansfield, MA, USA) 7×60 mm in external iliac artery] and two lesions requiring an endarterectomy of the femoral bifurcation. Three restenosis occurred (two diabetic patients) after 12 months of follow – up which were treated with a second DCB. Two patients underwent major lower limb amputations, both suffering from diabetes. Three patients died and one was lost during follow – up.

## VII. DISCUSSION

Restenosis after PTA or stenting of the femoro-popliteal tract is a growing and challenging problem. The disease is histologically characterized by the marked presence of fibrin deposition and accumulation of macrophages and multinucleated foreign body giant cells as compared to coronary and carotid atherosclerotic disease, which are richer in necrotic core (9,10) The first randomized, controlled trials (RCTs) comparing DCB with Paclitaxel vs standard uncoated PTA were THUNDER (11), PACIFIER (12), FemPac (13) and LEVANT I (14). All showed greater effectiveness and safety of DCB over POBA and reported that significant improvements of clinical parameters compared to preoperative values had taken place after 6 and 12 months (Rutherford classification and ankle-brachial index). Predominance of DCB over PTA alone was also confirmed by these trials in a large meta-analysis study (15).

Others clinical trials, LEVANT II study (Lutonix Paclitaxel – Coated Balloon for the Prevention of Femoropopliteal Restenosis) (5) and the IN.PACT SFA study (Randomized Trial of IN.PACT Admiral Drug Coated Balloon vs Standard PTA for the Treatment of SFA and Proximal Popliteal Arterial Disease) (6), evaluated two different DCBs. These balloons differ from each other in terms of excipient, paclitaxel dose and coating formulation. The Lutonix DCB with polysorbate/sorbitol and a 2 μg/mm^2^ dose of paclitaxel showed a primary patency of 73.5% at 12 months, while the IN.PACT Admiral DCB with urea and a paclitaxel dose of 3.5 μg/mm^2^ showed a primary patency of 86.6% at 12 months.

Recently published studies are the single – arm ILLUMENATE First-In-Human Study (16) and the ILLUMENATE Pivotal study (7) (prospective, randomized, single blind and multi-center study). The Pivotal study reported a primary patency per Kaplan – Meier estimates of 82.3% at 12 months in the DCB cohort vs 70.9% in PTA cohort. The mean length of lesions treated in this study is comparable with the main studies: THUNDER (7.4 cm), LEVANT I (8.1 cm), PACIFIER (7 cm) and IN.PACT SFA (8.94 ± 4.89cm).

In our study occlusions were found to be 18% higher rates than in the ILLUMENATE FIH study (12.1%) but lower than in the other main studies mentioned above. All occluded lesions were patent through 12 months, which suggests adequate effectiveness of DCB in CTOs. On the contrary, in our study primary patency was 93.9% at 12 months, which is better than the LEVANT 2 study (5) (73.5%) and comparable to the IN.PACT SFA study (6) (86.6%), ILLUMENATE FIH study (16) (89.5%) and ILLUMENATE Pivotal study (7) (82.3%). In our own study stents were only used in bail-out situations such as in flow – limiting dissections (stenosis > 50%) which happened in four cases. Calcifications on DCB effectiveness has been reported in the literature (17) yet definitions of calcium severity are found to differ as calcium may act as a barrier to the absorption of paclitaxel and circumferential calcium has been shown to be a stronger predictor of failure than longitudinal calcifications (18). Nevertheless, it is recognized that calcified and longer lesions remain a challenging subset that is less responsive to DCBs, determining a high rate of provisional stents (19). Directional atherectomy (DA) removes plaque from the vessel wall providing improved luminal gain and plaque modification resulting in low rates of bail-out stenting, perforation, and dissection (19).

The combination of DA plus DCB has been demonstrated to be safe and effective for long calcified lesions compared to treatment with DCB alone, however this has not yet been fully proven with an adequately powered randomized trial (20).

In conclusion treatment with DCB is safe and effective and can be considered a first choice technique in non-complex *de novo* lesions of the femoro – popliteal tract (TASC B and C lesions). Differences across DCBs can be explained by technical features such as drug dose, coating formulation, and excipient. These features impact drug tissue release timing and maintenance of therapeutic levels, as well as drug wash-out and downstream embolization. Procedural variables impact the effectiveness of the DCB. (7) The Stellarex DCB uses a 2 μg/mm^2^ paclitaxel dose combined with a polyethylene glycol (PEG) excipient. PEG is a polymer characterized by high molecular weight that results in durability of the drug coating, enabling it to be resistant to balloon deformation such as flexion and elongation. The combination of the hybrid coating and the durability of PEG is the basis of the drug transfer efficiency of the Stellarex DCB; the affinity of PEG for hydroxyapatite may maintain this transfer efficiency in the presence of calcified lesions and may explain the statistically superior primary patency of the Stellarex DCB over PTA (22), (23)

This study presents several limits: lack of a control arm, monocentric and retrospective study and the exclusion of post-PTAs and intrastent restenosis. But, our study, based upon mid-term/21-month data, reveals a low rate of restenosis and all a year following procedure, thereby extending the time to restenosis. Longer-term follow-up studies are recommended to ensure consistency of current data.
